# Impact of a health alert and its implementation on flutamide prescriptions for women: an interrupted time series analysis

**DOI:** 10.1186/s12913-020-05453-6

**Published:** 2020-06-29

**Authors:** Raquel Vázquez-Mourelle, Eduardo Carracedo-Martínez, Adolfo Figueiras

**Affiliations:** 1 Deputy Directorate-General Galician Health Service (Servicio Gallego de Salud - SERGAS), Galicia Regional Authority, Edificio Administrativo San Lázaro s/n, 15703 Santiago de Compostela, Galicia Spain; 2 Santiago de Compostela Health Area Authority, Galician Health Service, Santiago de Compostela, Galicia Spain; 3 Department of Preventive Medicine and Public Health, Faculty of Medicine, University of Santiago de Compostela, Consortium for Biomedical Research in Epidemiology & Public Health (CIBER en Epidemiología y Salud Pública - CIBERESP), Santiago de Compostela, Galicia Spain; 4 Health Research Institute of Santiago de Compostela (IDIS), Santiago de Compostela, Galicia Spain

**Keywords:** Flutamide, Off-label use, Patient safety, Drug misuse

## Abstract

**Background:**

Off-label drug use among ambulatory patients is often based on little or no scientific support. This paper reports the impact of a health warning about the risks of off-label flutamide use by women and the actions subsequently implemented by the public health service targeting such use.

**Method:**

The study was undertaken in a region in north-west Spain. We designed a segmented regression model of an interrupted time series, in which the dependent variable was the monthly value of defined daily doses of flutamide per 1000 inhabitants/day (DDD/TID), both total and stratified by sex. The following two data sources were used: flutamide prescriptions billed to the Spanish National Health Service; and flutamide deliveries made by wholesale drug distributors to pharmacies. The intervention assessed consisted of the issue of an official health warning and the actions subsequently taken to implement it.

**Results:**

There was an immediate reduction of 49.33% in DDD/TID billed to the Spanish National Health Service in respect of women; the mean value of the population percentage of DDD/TID of flutamide billed in respect of women fell from 34.4% pre-intervention to 23.72% post-intervention. There was an immediate reduction of 19.92% (95%CI: 6.68–33.15%) in total DDD/TID invoiced. There were no significant changes in DDD/TID billed in respect of men or in flutamide use in the private medical sector.

**Conclusions:**

Off-label drug misuse is a reality among ambulatory patients, even after actions are implemented following a toxicity warning issued by the competent Health Authority.

## Introduction

Flutamide is an oral, nonsteroidal anti-androgen drug, whose only authorised indication is treatment of metastatic carcinoma of the prostate [1]. Aside from a warning about flutamide hepatotoxicity, the Food and Drugs Administration’s label (product information sheet) on flutamide also states that “*Flutamide is for use only in men. This product has no indication for women, and should not be used in this population, particularly for non serious or nonlife-threatening conditions*” [2]. Nevertheless, there have been reports of off-label use of this drug in women to treat polycystic ovary syndrome, androgenetic alopecia, hirsutism, acne and seborrhea [3]. Evidence has been found of a high incidence of the side effects of flutamide, mainly hepatotoxicity, which appears to be especially significant among women [3].

Off-label drug use among ambulatory patients is often based on little or no scientific support [4], something that compromises patient safety and increases healthcare costs [4–7]. Its prevalence in European Union countries ranges from 6 to 72% [4]. There are studies which conclude that off-label use of a given drug leads to a higher incidence of adverse reactions than does its on-label use [7].

In April 2017, the Spanish Agency of Medicines and Medical Devices (*Agencia Española de Medicamentos y Productos Sanitarios/AEMPS*) issued an informative health warning, alerting to the problems of flutamide use and advising against its off-label use by women, due to associated risks of hepatotoxicity [8]. The following month the public health service of one Spain’s Autonomous Regions sought to implement this warning by suspending all electronic prescriptions of flutamide for women, accompanied by a notice sent to the prescriber. To our knowledge, no other study has analysed the impact of an official health authority warning on the prescription and use of flutamide. Accordingly, the designated aim of this study was to assess the impact of the *AEMPS* warning on the above Spanish Region and the regional health authority actions implemented in response to this health warning about off-label flutamide use by women.

## Materials and methods

### Scope

The study is the result of an internal control programme developed in 2017 in Galicia, a region in the north-west of Spain with a population of 2.7 million, 98.4% of which is covered by the Spanish National Health Service (SNHS) under a public health insurance system [9]. Out-of-hospital use of drugs prescribed by SNHS physicians is subject to a financial contribution (co-payment).

In the Galician Autonomous Region there are 23 wholesale drug distributors which supply the area’s 1349 retail pharmacy outlets. The SNHS employs 6400 prescribing physicians via the regional public health service [10].

When the *AEMPS* authorises a drug, it does so accompanied by a legally valid document, setting out the therapeutic indications, dose, contraindications, adverse reactions and/or efficacy. This is known as the “*ficha técnica”* or *“resumen de características del producto*”; the European Medicines Agency equivalent is called Summary of product characteristics, and the Food and Drug Administration equivalent is called “Label, product information sheet or Prescription Drug Labeling” [11] .

Prior to 2009, off-label drug use in Spain, as in many other European countries, required prior authorisation by the state drug agency [12, 13]. Currently such use must be of an exceptional nature, be limited to those situations in which no approved therapeutic alternatives exist, be supported by sufficient scientific evidence, and be accompanied by the patient’s informed consent and the approval of the committee responsible for treatment protocols in each Autonomous Region [13, 14] .

In 2017, the *AEMPS* issued a health warning, due to severe cases of hepatotoxicity related to off-label flutamide use among women [8].

### Study design and data sources

We conducted a quasi-experimental ecological time-series study, using monthly drug usage data from January 2014 to December 2017.

The total information analysed corresponded to:

- deliveries made by all wholesale drug distributors to retail pharmacy outlets in the region;
- invoices of officially prescribed prescriptions submitted by retail pharmacy outlets to the SNHS in the region via the official administrative reporting systems; and,
- use corresponding to the private medical sector, obtained by subtracting retail pharmacy invoicing from total deliveries above, i.e., (b)-(a). Under Spanish Law, retail pharmacies may only sell medicines to individual patients (and not, for instance, to wholesale companies), while individual patients may obtain prescriptions from either the public or the private sector, with flutamide being classed as a drug available exclusively on prescription.

To calculate monthly use, we first calculated the number of Defined Daily Doses (DDD), assuming a DDD of flutamide to be 750 mg [15]. Once transformed into the number of DDD per 1000 inhabitants per day (DDD/TID) [16, 17], these are the basic units that enable time sequences to be examined, adjusting for potential changes in the population, dosage, and/or number of pills per prescription or package. The number of DDD/TID also makes it possible to ascertain the prevalence of utilisation of a given drug. For instance, use of 20 DDD/TID in a given month can be interpreted to mean that on each day of that month an average of 20 of every 1000 inhabitants had taken one DDD, namely, that about 2% of the population is taking the medicine in question [18].

### Ethical aspects

This study was conceived as an internal audit, aimed at verifying the result of or compliance with actions implemented by the Public Health Service itself. Official billing data sources are capable of automatically supplying auditors with DDD data stratified by men and women. There was thus no need to set up or directly manage databases in which there might have been identifiable or identified patient data. All this meaning that according to local regulations obtaining an ethics committee approval to carry out this study was not necessary.

### Interventions

The following two consecutive actions were evaluated as a single intervention: (1) the informative health warning issued by the *AEMPS* in April 2017 [8]; and, (2) its implementation the following month by means of suspending active flutamide prescriptions for women in the SNHS regional service electronic prescription system; this suspension takes the form of a notice about the health warning which is automatically sent to a prescribing physician when a flutamide prescription is generated for a woman patient, with the aim of convincing him/her to reconsider and cancel it. Even so, the physician may re-activate the prescription, overriding the warning. The notice contains the following message, “*Evaluate the continuity of flutamide treatment in accordance with the security warning of the Spanish Agency of Medicines and Medical Devices* (https://www.aemps.gob.es/informa/notasInformativas/medicamentosUsoHumano/seguridad/2017/docs/NIMUH_FV_032017flutamida.pdf).”

### Statistical analysis

For statistical analysis purposes, we constructed a segmented regression model of the interrupted time series for each independent variable analysed [19–22]. In this analysis, the dependent variable was DDD/TID per month, both total and stratified by sex; and the independent variables were:

- time from study onset, which reflects the time trend prior to the study intervention;
- a binary variable which took a value of 0 before and a value of 1 after the intervention, and would thus show the immediate change to each; and,
- a temporal variable which took a value of 0 before and a value of 1,2,3 after the intervention, and would thus show the long-term change in the trend.

To this end, we used the equation described by Linden [19].

In order to reduce any possible autocorrelation, we introduced autoregressive terms into the two models.

Given that the two interventions were consecutive (one in April and the other in May 2017), for statistical calculation purposes April and May 2017 were deemed to be a transition period, with the impact of the two interventions being analysed as one single intervention**,** by taking the months prior to April 2017 as the pre-intervention period, and the period subsequent to May 2017 as the post-intervention period.

## Results

### Pre-intervention period

**Fig. 1:**
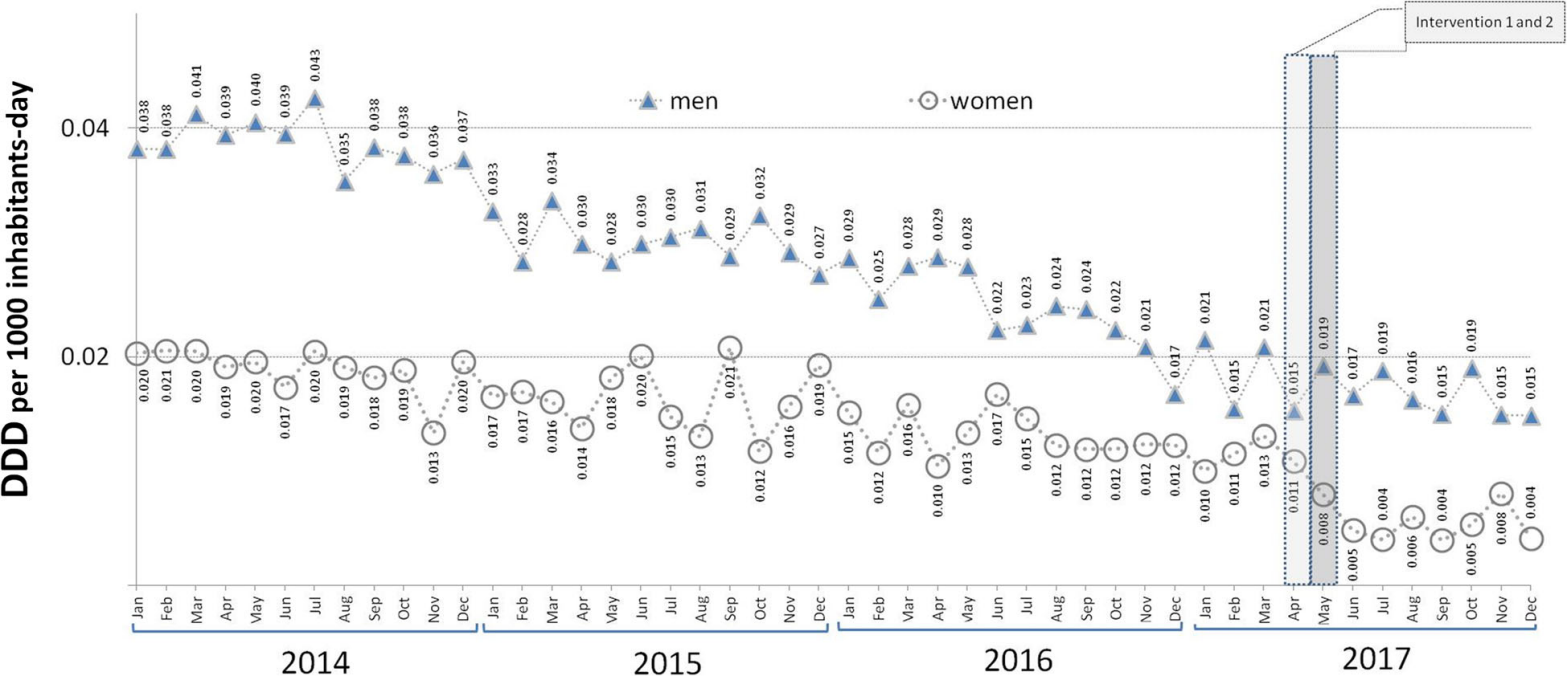
Trends in defined daily doses per 1000 inhabitants per day (DDD/TID) of flutamide billed to the Spanish National Health Service via the public regional health service

**Fig. 2:**
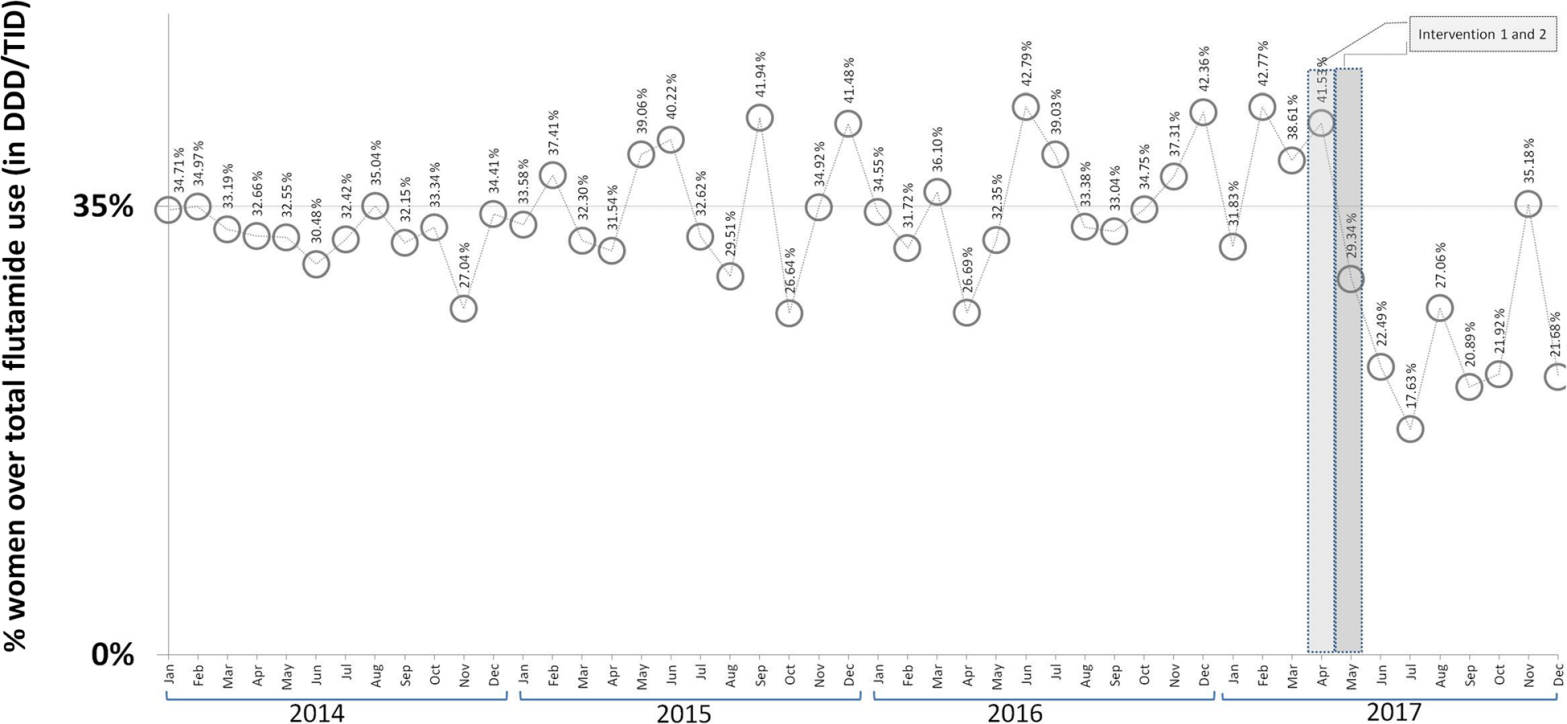
Trend in the percentage of defined daily doses per 1000 inhabitants per day (DDD/TID) of flutamide in women with respect to total DDD/TID of flutamide consumed

**Fig. 3:**
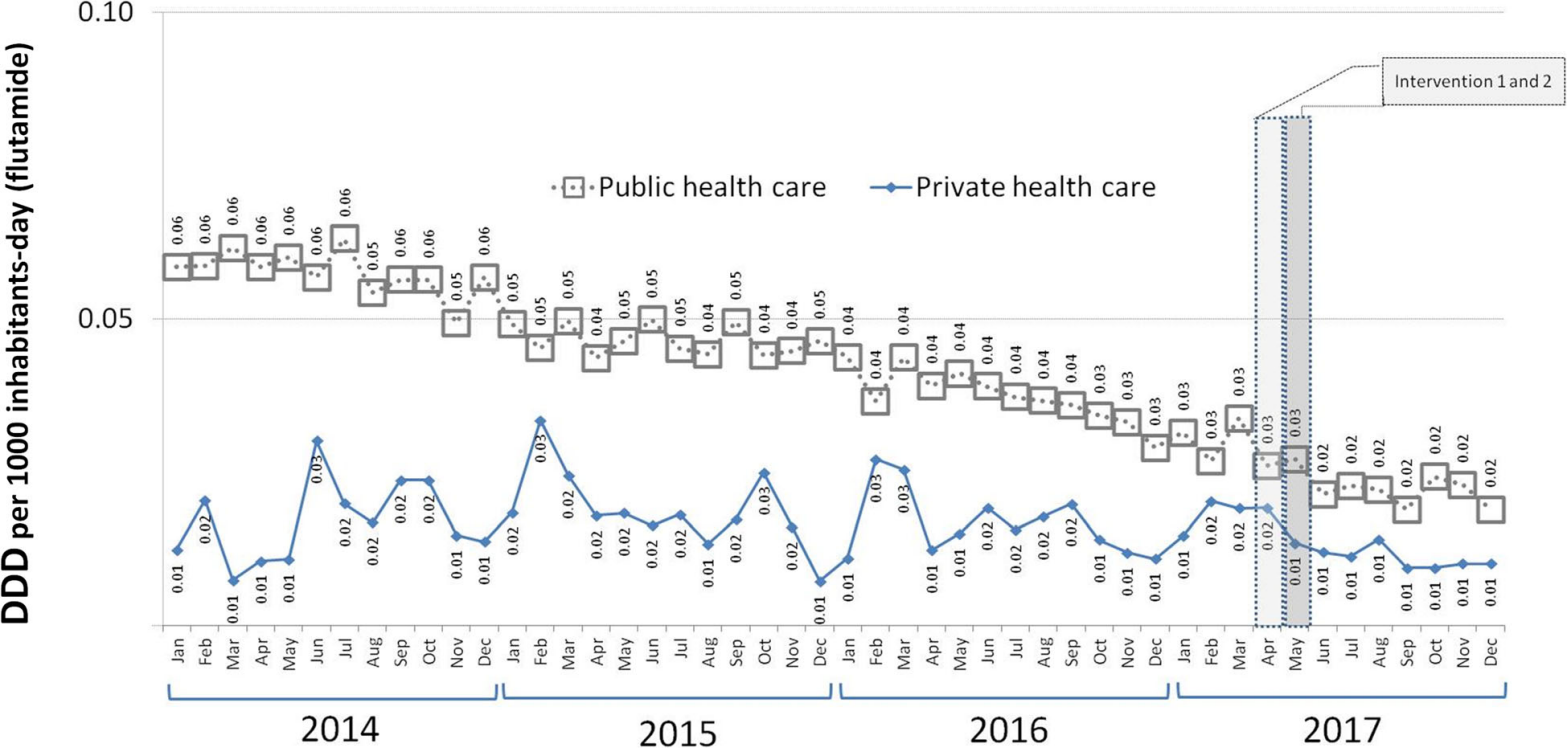
Trends in flutamide use in public and private health care, expressed in Defined Daily Doses per 1000 inhabitants per day (DDD/TID)

The results for the segmented regression show that in the pre-intervention period, the SNHS trend in flutamide prescriptions was downward for both men (annual decrease of 17.46%, *p* < 0.05) and women (annual decrease of 16.61%, p < 0.05) (see Table 1 and Fig. 1). The slope of this decrease in men was double that of women, which translates as a rise in the percentage of women’s prescriptions with respect to the total (4.93%; *p* < 0.05) (Fig. 2). During the pre-intervention stage, no downward trend in prescriptions was observed outside the ambit of the SNHS (see Table 1 and Fig. 3).

**Table 1:** Segmented regression of time-series data expressed in doses per 1000 inhabitants per day (DDD/TID) of flutamide

FLUTAMIDE	Pre-intervention trend		Post-intervention trend			
			Immediate impact		Long-term change in trend	
	Coefficient^a^	95% CI	Coefficient^b^	95% CI	Coefficient^c^	95% CI
DDD/TID in women billed to SNHS	−0.00028*	− 0.00044 to − 0.00012	− 0.00610*	− 0.00912 to − 0.00308	0.00004	− 0.00050 to 0.00058
DDD/TID in men billed to SNHS	−0.00059*	−0.00065 to − 0.00053	0.00283	−0.00076 to 0.00642	−0.00058	−0.00117 to 0.00001
Sum of DDD/TID for women and men billed to SNHS	−0.00082*	−0.00090 to − 0.00074	−0.004176*	−0.00804 to −0.00031	0.00082	−0.00045 to 0.00101
% DDD/TID billed for women with respect to total DDD/TID billed	0.14262*	0.02488 to 0.25965	−13.10626*	−18.85022 to −7.36230	−0.24261	−1.19333 to 0.70811
DDD/TID use in healthcare outside the ambit of the SNHS	−0.00003	−0.00018 to 0.00012	− 0.00269	−0.01055 to 0.00517	− 0.00114	−0.00283 to 0.00055

* *p* < 0.05

SNHS: spanish national health system via the public regional health service

^a^β_1_: slope of the dependent variable until the introduction of the intervention

^b^β_2_: change in the level of the dependent variable, which occurs in the period immediately after versus the period immediately before the introduction of the intervention

^c^β_3_: difference between the pre- and post-intervention period in the slope of the dependent variable

### Immediate effect

On evaluating the effect of the interventions on the immediate reduction in flutamide use, SNHS prescriptions among women were observed to decrease by 34.09% (*p* < 0.05), whilst in men no immediate effect was to be seen (see Table 1 and Fig. 1). The effect on women means that overall prescriptions (sum of men and women) also decreased by 19.92% (*p* < 0.05). This effect of the intervention on total prescriptions was not in evidence outside the ambit of the SNHS (*p* > 0.05) (see Table 1, and Fig. 3).

From the prevalence of flutamide utilisation by women, it is estimated that prescribers were sent 30 electronic alerts, 13 of which were overridden.

### Long-term effect

Assessment of the mean monthly population value of all SNHS flutamide prescriptions accounted for by women shows that it fell from 34.40% prior to the intervention to 23.72% during the post-intervention period. The intervention was not observed to have brought about a change in the long-term slope of any of the study variables (see Table 1).

## Discussion

This study shows that the joint effect of the AEMPS safety warning and the subsequent e-prescription system suspension of active flutamide prescriptions in women was to cut the amount of DDD/TID prescribed in the Galician Health Service by half. This indicates that: (1) there had been prior misuse [2, 3, 8]; and (2) that the Public Health Service actions were effective. Despite the warning issued about the risks of hepatotoxicity, this population group nonetheless maintained a level of flutamide use equivalent to 20% of the total amount consumed.

Although we were unable to locate any previous study on the impact of a health warning regarding the specific risks of off-label drug use, we did manage to locate studies on the impact of drug health alerts regarding general risks or on-label use, which reported varying results [23–27].

The short space of time which elapsed between the *AEMPS* warning and the subsequent SNHS regional health service suspension of electronic prescriptions in women meant that the statistical method used could not identify the separate impact of each intervention. However, since the SNHS suspension exclusively affected women in the SNHS, a detailed study by sex made it possible to assess the impact of each of the interventions. Hence, the immediate effect among women was clear, with a reduction to almost half; among men, however, there was no significant change after the health alerts. This could indicate that the impact of the health warning was relatively lower, whereas that of the suspension was relatively higher.

The finding that the suspension had a greater effect than the alert is further supported by the fact that, following the interventions, total flutamide use underwent an immediate reduction in the SNHS (where a suspension of electronic prescriptions was in place in respect of women), while in healthcare outside the ambit of the SNHS (where no such suspension took place) this did not occur.

The results also indicate that, during the pre-intervention period, there was a rise in the percentage of women who engaged in off-label flutamide use. This fact alone justifies the need for a specific safety warning for this group.

Our study observed that, despite the health warning and suspension of active electronic prescriptions, off-label flutamide use continued. This may be due to:

(i). off-label pharmaceutical marketing [28], though illegal and potentially subject to sizeable penalties [29];
(ii). prescribers’ ignorance of prescribing obligations. Off-label drug use is only legal, if each and every one of a series of requirements is met, i.e., existence of sufficient scientific evidence, lack of other approved therapeutic alternatives, approval by a regional committee [13, 14], and the patient’s informed consent [30]. Failure to comply with these requirements amounts to an unlawful act, which gives rise to disciplinary repercussions as well as the related healthcare and economic harm [5];
(iii). pressure resulting from complacency towards the patient, with patient pressure having been seen to induce drug prescribing [31]; and/or,
(iv). maintenance of state funding in the absence of clear explicit statutory provisions which would expressly exclude off-label use from public funding in Spain, despite the fact that theoretically, off-label use is not reimbursed [4–6].

To decrease the use of this drug, measures with proven effectiveness could be implemented, such as: (a) submitting the dispensing of flutamide to a prior authorisation requirement [31–36]; (b) introducing drug-inspection programmes [37, 38]; or, (c) returning Spain to the system that is still in place in other European countries, whereby off-label use must be previously authorised by the state drug agency [4].

This study’s limitations would arguably include its ecological design or the lack of a control group, though, as other potentially related external factors should have affected both sexes equally, the men’s group could be regarded as a control group for the women. A further limitation might lie in our not having information about possible deliveries made directly by pharmaceutical companies or authorised wholesale drug distributors outside the study region, though use of this circuit is not standard practice. Lastly, the two actions (the issue of the health warning and its subsequent implementation) were very close in time, thereby rendering it impossible to assess their respective effects separately.

## Conclusion and implications

Off-label misuse of drugs is a reality among ambulatory patients, even after the issue of a toxicity warning by the state regulatory agency. Accordingly, the implementation of strict control programmes in health services is called for, as well as a specific, clear regulation in line with the necessary degree of healthcare safety required by off-label use.

## Data Availability

The datasets used and/or analysed during the current study are available from the corresponding author on reasonable request.

## Abbreviations

AEMPS: Spanish Agency of Medicines and Medical Devices
SNHS: Spanish National Health Service
DDD: Defined daily doses
DDD/TID: Defined daily doses per 1000 inhabitants per day
DDD/100 s-d: Defined daily doses per 100 stays and a day
